# Sulfonylureas Use Is Not Associated With Increased Infarct Size in Patients With Type 2 Diabetes and ST-Segment Elevation Myocardial Infarction

**DOI:** 10.3389/fcvm.2021.658059

**Published:** 2021-05-28

**Authors:** Fang-Hong Shi, Hao Li, Ling-Cong Kong, Long Shen, Yi-Hong Jiang, Zhi-Chun Gu, Heng Ge

**Affiliations:** ^1^Department of Pharmacy, Renji Hospital, School of Medicine, Shanghai Jiaotong University, Shanghai, China; ^2^Department of Cardiology, Renji Hospital, School of Medicine, Shanghai Jiaotong University, Shanghai, China; ^3^Department of Pharmacy, Shanghai Children's Medical Center, School of Medicine, Shanghai Jiaotong University, Shanghai, China; ^4^Department of Endocrinology, Renji Hospital, School of Medicine, Shanghai Jiaotong University, Shanghai, China

**Keywords:** ST-segment elevation myocardial infarction, type 2 diabetes mellitus, sulfonylureas, infarct size, cardiac magnetic resonance, microvascular obstruction, heart failure

## Abstract

**Aims:** This retrospective study assessed the association between sulfonylureas use and infarct size in patients with type 2 diabetes (T2DM) and ST-segment elevation myocardial infarction (STEMI) by myocardial enzymology indexes and cardiac magnetic resonance (CMR) imaging.

**Methods:** Patients presenting STEMI between July 2013 and August 2019 were included in a retrospective database at our institution. Antidiabetic agents used before STEMI were recorded. Patients with maximum recorded troponin I (max cTNI) and creatine phosphokinase isoenzyme (CK-MB) within the first 72 h of chest pain onset were selected. Infarct size was quantified by CMR imaging, and cardiovascular outcomes were also obtained at 30 days and 6 months follow-up. Multivariable regression models explored potential risk factors associated with infarct size and clinical outcomes.

**Results:** A total of 254 T2DM and STEMI patients were included, with 101 sulfonylurea users and 153 non-users. Sulfonylureas users were not associated with higher max cTnI and max CK-MB compared to non-users. Among 65 CMR patients, no significant differences in infarct size were detected between sulfonylureas users and non-users. Whereas, the incidence of microvascular obstruction (MVO) was higher in patients receiving sulfonylureas than those taking non-sulfonylureas (88.0 vs. 62.5%, *p* = 0.023). No higher cardiovascular events of sulfonylureas users vs. non-users were observed, except for heart failure events (24.0 vs. 2.5% at 30 days, *p* = 0.011; 28.0 vs. 2.5% at 6 months, *p* = 0.004). Multivariable regression analyses verified that sulfonylureas users increased the risks of MVO.

**Conclusions:** Sulfonylureas use did not associate with larger infarct size in patients with T2DM and STEMI. A potentially higher incidence of MVO in sulfonylurea users was found. Notably, since most patients presented after a relatively long period of ischemia and glibenclamide was not used by the included patients in this observational study, the results of this study should not be extrapolated to clinical settings with short periods of ischemia or to patients using glibenclamide.

**1. What is already known about this subject:**

There is controversy over whether sulfonylureas associates with larger infarct size in diabetic patients with ST-segment elevation myocardial infarction (STEMI).

**2. What this study adds:**

Our study's major advantage was to determine infarct size by cardiac magnetic resonance techniques. We found that sulfonylureas use did not associate with higher infarct size in patients with T2DM and STEMI assessed by both myocardial enzymology indexes and cardiac magnetic resonance imaging. Potentially higher incidence of microvascular obstruction in sulfonylurea users was found.

## Introduction

Type 2 diabetes mellitus (T2DM) is a severe metabolic condition characterized by relative insulin deficiency caused by pancreatic β-cell dysfunction and insulin resistance (1). Cardiovascular disease is the major macrovascular complication of T2DM and increases a higher mortality risk than patients without cardiovascular disease (2, 3). Diabetes mellitus (DM) is a significant risk factor for acute myocardial infarction (AMI) and about 30% comorbidity in patients hospitalized with AMI. The presence of DM doubles the mortality during both the acute phase of AMI and at long-term follow-ups (4). Currently, sulfonylureas are still used frequently as second-line treatment in patients with T2DM because they effectively improve glycemic control and reduce HbA1c levels by 1.0–1.5% (1, 5, 6). In China, ~34% of diabetic patients are initial treated with sulfonylureas, and the leading commercially available sulfonylureas are glimepiride, gliclazide, glipizide, gliquidone, and glyburide (5, 7). However, the extensive clinical application of sulfonylureas has raised concerns on the risk of adverse cardiovascular events, initially presented in the University Group Diabetes Program (UGDP) study in 1970 (8). In animal studies, sulfonylureas were believed to disrupt the protective effects of ischemic conditioning and subsequently increase infarct size and reduce left ventricular function (9, 10), while two recent randomized controlled trials comparing sulfonylureas with either pioglitazone (TOSCA trial) or linagliptin (CAROLINA trial) failed to observe a higher risk for cardiovascular events with sulfonylureas treatment (11, 12). Conflicting findings from the above studies have fueled the controversy on a possible relationship between sulfonylureas use and larger myocardial infarct size. Besides myocardial enzymology indexes, cardiac magnetic resonance (CMR) imaging is a more appropriate and precise method to quantify infarct size in patients with ST-segment elevation myocardial infarction (STEMI) (13, 14). The use of infarct size determination by CMR techniques has not evaluated whether sulfonylureas use affects STEMI patients. Therefore, we sought to use myocardial enzymes and CMR imaging to investigate the association between sulfonylureas use and infarct size in T2DM patients presenting with STEMI.

## Methods

### Study Population

First-time STEMI patients admitted to our institution from July 2013 to August 2019 were included if they had a history of T2DM and received long-term antidiabetic treatment and had at least three records of troponin I (cTNI) and creatine phosphokinase isoenzyme (CK-MB) within 72 h of chest pain onset. The clinical lab never changed the detection method of cTnI and CK-MB from 2013 to 2019 [cTNI Kit: BECKMAN COULTER, Registration Code (China): 20202400025; CK-MB Kit: Johnson & Johnson, Registration Code (China): 20152402813]. Of multiple records, the maximum cTNI and CK-MB were included in our analysis. If patients were admitted to our institution several times for STEMI during our observation period, only the first admission was included. The exposure of interest was long-term treatment (at least 6 months) of sulfonylureas before STEMI, which was defined as the documentation of prescriptions for sulfonylureas (gliclazide, glimepiride, glipizide, gliquidone, etc.). Non-sulfonylureas therapy was considered the prescription information for other antidiabetic agents (metformin, alpha glycosidase inhibitors, thiazolidinediones, glinides, insulin, dipeptidyl peptidase-4 inhibitors, etc.). Among the included population, patients who received the primary percutaneous coronary intervention (PCI) within 12 h after symptom onset and underwent CMR examination within the first week of STEMI onset were considered a CMR population. The exclusion criteria for a CMR examination include (1) physical instability for the examination, (2) previous implantation of non-CMR-conditioned metal devices, (3) claustrophobia, and (4) personal refusal. The ethics committees of Renji Hospital approved the study protocol (No. KY2019-159).

### CMR Imaging and Analyses

**Figure 1:**
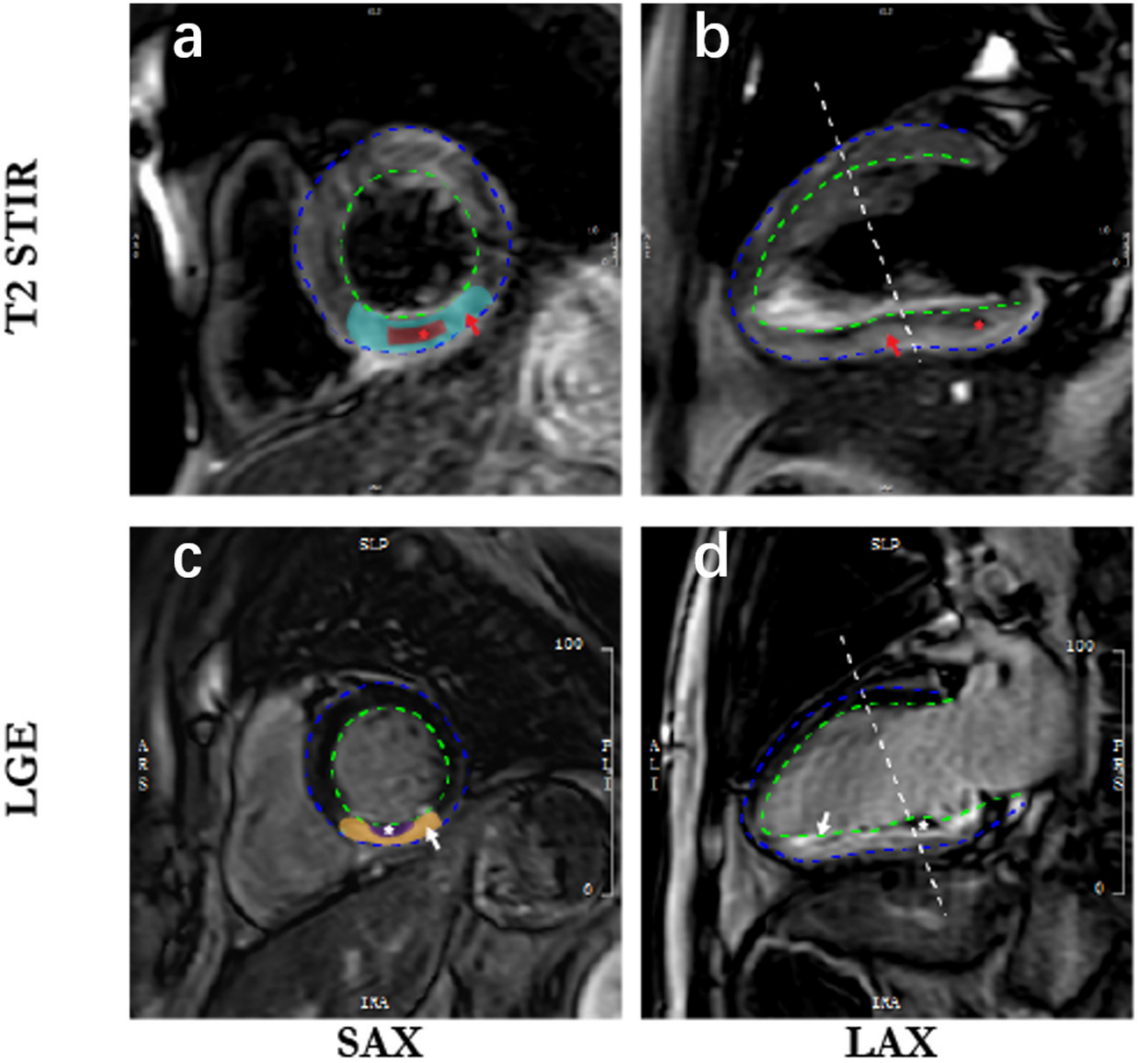
Cardiac magnetic resonance imaging in patients with acute myocardial infarction. SAX, short axis; LAX, long axis; LGE, late gadolinium enhancement. The blue dotted line is epicardium; the dotted green line is endocardium; **(a)** indicates myocardial edema (blue-green zone) defined as high-signal myocardium within the territory of the culprit's vessel; **(b)** shows the intramyocardial hemorrhage (red star) in the hyposignal region within the edema; **(c)** is infarction (yellow zone) defined as hyperenhanced myocardium, and corresponding infarct size is quantified and normalized to the left ventricle mass; 1 **(d)** represents microvascular obstruction (white zone) defined as a hypo-enhanced area within the infarcted-related zone.

Electrocardiographically gated CMR imaging was performed using a 3.0-Tesla scanner (Achieva TX, Philips Healthcare, Best, The Netherland) within 7 days after the first chest pain onset. All sequences were acquired in breath-hold, with a field of view of 350 × 350 mm^2^. An experienced reader (HG) analyzed the CMR results by using commercial software (QMass MR 7.5, Medis Medical Imaging, Leiden, The Netherland). Cine CMR was performed using a balanced steady-state free precession sequence in a short-axis view to cover the whole left ventricle (LV) without a gap (repetition time/echo time, 3.2/1.6 ms; 30 phases; voxel size, 2.0 × 1.6 × 8 mm^3^). Upon a black-blood T2 short-tau inversion-recovery imaging (T2W-STIR; repetition time/echo time, 2 R-R intervals/75 ms; voxel size, 2.0 × 1.6 × 8 mm^3^), myocardial edema was defined as high-signal myocardium within the territory of the culprit's vessel (signal intensity > 2 SDS above the mean signal in remote non-infarcted myocardium) (Figure 1a), and the hyposignal region within the edema was recognized as intramyocardial hemorrhage (IMH, which is a sign of microvasculature rupture) (Figure 1b). Based on late gadolinium-enhanced imaging (3D inversion recovery segmented gradient echo sequence 10 min after contrast injection at short-axis and 2-, 4-chamber views covering the whole LV; repetition time/echo time, 3.5/1.7 ms; temporal resolution, 190 ms; voxel size, 1.5 × 1.7 × 10 mm^3^ interpolated into 0.74 × 0.74 × 5 mm^3^), infarction was defined as hyperenhanced myocardium with a signal intensity >5 SDS of the nulled remote myocardium (Figure 1c), and microvascular obstruction (MVO) was defined as a hypoenhanced area within the infarcted-related zone (Figure 1d). Ventricular volumes and left ventricular ejection fraction (LVEF) were calculated based on short-axis slices of cine images covering the whole heart. Myocardial masses within the software's contours were provided, and the extent of both infarction and MVO were semiquantified as a percentage of left ventricular myocardial mass (% LVM). In this study, we did not use the area at risk as a measurement for the following reasons: (1) the scanning sequence is relatively slow and requires breath-hold for 15–20 s to image a single slice. Considering the heart function of STEMI patients, it is not safe to perform multiple-slice scanning to cover the whole LV like the LGE scanning; (2) just like the measurement of LGE, the outline of the “area at risk” is based on a self-contrast between healthy myocardial and myocardial with edema. However, the contrast is not as apparent as in LGE images, which use gadolinium to enhance the contrast. Therefore, we performed edema imaging in most study participants based on a three-slice strategy to visualize intramyocardial hemorrhage. Still, these data are not reliable to provide the accurate area at risk.

### Outcomes Measures

For the total population, the primary outcome variables were max cTnI, max CK-MB, max CK, and the secondary outcomes were LVEF and brain natriuretic peptide (BNP) within the first 72 h of chest pain onset. For the CMR population, the primary results were infarct size (expressed as % LVM), and the secondary outcomes included MVO and IMH detected by CMR imaging.

### Follow-Up

Follow-up was performed at 30 days and 6 months post-STEMI. Major cardiovascular adverse events (MACEs) were defined as a composite of cardiovascular death, recurrent myocardial infarction (MI), non-fatal stroke, and recurrent angina. Other outcomes included individual cardiovascular events (cardiovascular death, recurrent MI, non-fatal stroke, recurrent angina, revascularization, heart failure) and bleeding. Cardiologists reviewed all available information and used their clinical expertise to adjudicate the cause of death. Categories of cardiovascular death include sudden death, worsening of heart failure, acute myocardial infarction, stroke, cardiogenic shock, or other cardiovascular death (15). Heart failure includes heart failure with preserved and reduced ejection fraction. The criteria of heart failure mainly include symptoms with or without signs, LVEF preserved or reduced, elevated levels of BNP, and at least one additional criterion of the following: (1) relevant structural heart disease or (2) diastolic dysfunction (16).

### Statistical Analysis

Continuous variables were described as mean with standard deviation and compared by unpaired Student's *t* tests or Mann–Whitney *U* tests. Categorical variables were described as the number and percentage and compared by chi-square test or Fisher's exact tests. A multivariable linear regression model was used to explore the potential risk factors associated with infarct size. Besides, single and multivariable logistic regression models were used to determine the independent risk factors for MVO and heart failure. Two criteria were considered necessary for a variable to be entered in the multivariable analysis model: (1) a univariate *P* value for the risk of MVO and heart failure ≤0.10 and (2) a plausible association with the risk of MVO and heart failure in patients with STEMI according to data provided by the literature. To robust the results, further subgroup analyses were performed by only including patients who have anterior infarction or whose symptom-to-balloon time was <12 h. All analyses were performed using the SPSS software, version 22.0 (SPSS Inc., Chicago, Illinois, USA), and *P* < 0.05 was considered significant.

## Results

### Patient Characteristics

**Figure 2:**
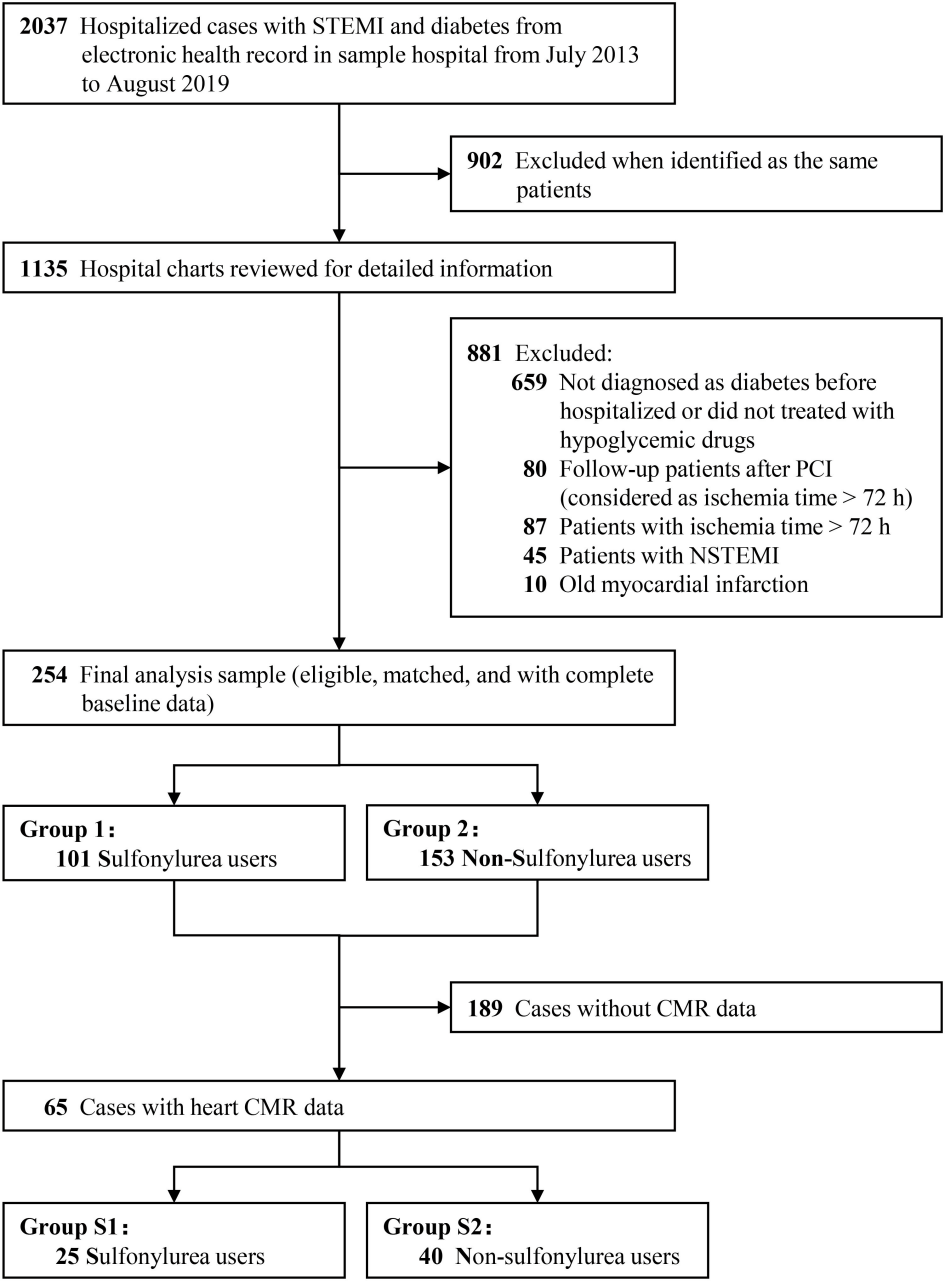
The flow diagram of the selection process to determine eligible individuals. STEMI, ST-segment elevation myocardial infarction; NSTEMI, non-ST-segment elevation myocardial infarction; CMR, cardiac magnetic resonance imaging; PCI, percutaneous coronary intervention.

Figure 2 presents the flow diagram of the selection process to determine eligible patients. A total of 2,037 hospitalized cases with T2DM and STEMI from electronic health records were reviewed. Finally, 254 diabetic patients were eligible for inclusion criterion, with 101 sulfonylurea users and 153 non-sulfonylurea users. CMR imaging was performed in 65 patients to determine the infarct size, among which 25 patients received sulfonylureas and 40 patients received other antidiabetic agents. The proportion of sulfonylurea use in total and CMR population were 39.8 and 38.5%, respectively. Of the total population, the sulfonylureas taken by the patients included gliclazide (41.6%), glipizide (23.8%), glimepiride (31.7%), and gliquidone (3.0%). Among the CMR population, sulfonylureas use consisted of gliclazide (60%), glipizide (28.0%), and glimepiride (12.0%). Baseline characteristics of the total population were relatively comparable between sulfonylurea and non-sulfonylurea users, except for body weight mass index (BMI), diabetic duration, chronic kidney disease, anterior infarction, and insulin (*p* < 0.05 for each variable) (Table 1). Among the CMR population, the baseline characteristics between the two groups were entirely similar (Table 1). Moreover, compared with the total population, CMR population had more dyslipidemia or Killip class II–IV and shorter symptom-to-balloon time (Supplementary Table 1).

**Table 1:** Baseline characteristics of total population and CMR population.

	**Total population**		**CMR population**	
**Variables**	**SU group**	**Non-SU group**	**SU group**	**Non-SU group**
	**(*n* = 101)**	**(*n* = 153)**	**(*n* = 25)**	**(*n* = 40)**
Age, years	65.7 ± 9.84	65.5 ± 11.1	61.3 ± 7.4	61.3 ± 7.8
Male, *n* (%)	75 (74.3)	123 (80.4)	20 (80.0)	37 (92.5)
Body weight, mean ± SD	67.8 ± 8.5	72.6 ± 10.9^*^	68.0 ± 8.0	72.9 ± 12.0
BMI, kg/m^2^	23.8 ± 2.0	25.2 ± 3.4^*^	23.7 ± 2.1	25.0 ± 3.4
HbA1c%, mean ± SD	8.0 ± 1.6	8.0 ± 1.6	7.7 ± 1.5	8.0 ± 1.9
Diabetic duration, year	7.6 ± 4.9	9.5 ± 7.3^*^	6.8 ± 3.4	6.9 ± 4.7
Time to CMR imaging, days	/	/	5.1 ± 1.5	4.5 ± 3.0
**Cardiovascular risk factors**				
Hypertension, *n* (%)	71 (70.3)	111 (72.5)	15 (60.0)	29 (72.5)
Dyslipidemia, *n* (%)	18 (17.8)	31 (20.3)	15 (60.0)	22 (55.0)
Smoking, *n* (%)	20 (19.8)	39 (25.5)	12 (48.0)	28 (70.0)
Chronic kidney disease, *n* (%)	2 (2.0)	14 (9.2)^*^	0 (0)	1 (2.5)
Myocardial infarction history, *n* (%)	0 (0)	0 (0)	0 (0)	0 (0)
Killip class II-IV, *n* (%)	16 (15.8)	23 (15.0)	11 (44.0)	11 (27.5)
Anterior infarction, *n* (%)	58 (57.4)	65 (42.5)^*^	11 (44.0)	16 (40.0)
**Reperfusion management**				
PCI, *n* (%)	101 (100)	153 (100)	25 (100)	41 (100)
Symptom-to-balloon time, hour	13.3 ± 14.1	13.5 ± 13.9	5.7 ± 4.2	5.1 ± 2.0
**Preadmission drugs**, ***n*** **(%)**				
Gliclazide	42 (41.6)	/	15 (60.0)	/
Glipizide	24 (23.8)	/	7 (28.0)	/
Glimepiride	32 (31.7)	/	3 (12.0)	/
Gliquidone	3 (3.0)	/	0 (0)	/
Glibenclamide	0 (0)	/	0 (0)	/
Metformin	30 (29.7)	49 (32.0)	8 (32.0)	15 (37.5)
Acarbose	26 (25.7)	49 (32.0)	8 (32.0)	24 (60.0)^*^
Voglibose	2 (2.0)	5 (3.3)	0 (0)	1 (2.5)
Insulin	4 (4.0)	72 (47.1)^**^	1 (4.0)	8 (20.0)
Thiazolidinediones	4 (4.0)	7 (4.6)	0 (0)	1 (2.5)
other AHA	8 (8.0)	16 (10.5)	2 (8.0)	5 (12.5)
Calcium channel blocker	22 (21.8)	49 (32.0)	3 (12.0)	9 (22.5)
Beta-blocker	7 (6.9)	21 (13.7)	0 (0)	5 (12.5)
ACEI or ARB	31 (30.7)	41 (26.8)	3 (12.0)	3 (7.5)
Lipid lowering drug	11 (10.9)	19 (12.4)	4 (16.0)	10 (25.0)
Antiplatelet agents	32 (31.7)	43 (28.1)	14 (56.0)	24 (60.0)
Anticoagulants	1 (1.0)	3 (2.0)	1 (4.0)	1 (2.5)

*CMR, cardiac magnetic resonance; SU, sulfonylureas; STEMI, ST-elevation myocardial infarction; BMI, body mass index; HbA1c%, glycosylated hemoglobin; PCI, percutaneous coronary intervention; AHA, anti-hyperglycemic agents; ACEI, angiotensin converting enzyme inhibitors; ARB, angiotensin receptor blocker; SU group compared to non-SU group in total population and SU group compared to non-SU group in CMR population*.

* *Means p < 0.05 and*

** *Means p < 0.01*.

### Outcomes in Total Population

For total population, max cTnI [mean (SD): 28.9 (26.5) ng/ml vs. 32.7 (29.3) ng/ml, *p* = 0.268], max CK-MB [mean (SD):177.3 (171.9) ng/ml vs. 152.1 (160.8) ng/ml, *p* = 0.244], and max CK [mean (SD):1748.8 (1721.3) ng/ml vs. 1597.4 (1778.6) ng/ml, *p* = 0.499] was similar between sulfonylurea users and non-sulfonylurea users. In addition, as for heart failure indexes, LVEF [mean (SD): 51.8 (10.6)% vs. 52.0 (10.7)%, *p* = 0.898] and BNP [mean (SD): 463.6 (529.3) ng/ml vs. 555.5 (841.7) ng/ml, *p* = 0.288] did not differ between sulfonylurea users and non-sulfonylurea users (Table 2). Two subgroup analyses that included patients who had anterior infarction or whose symptom-to-balloon time was <12 h confirmed the robustness of primacy results (Supplementary Tables 2, 3).

**Table 2:** Association ofsulfonylurea use with cardiac enzymes and heart failure indexes.

	**Total population**		**CMR population**	
**Outcomes**	**SU group**	**Non-SU group**	**SU group**	**Non-SU group**
	**(*n* = 101)**	**(*n* = 153)**	**(*n* = 25)**	**(*n* = 40)**
Max Troponin I (ng/ml)	28.9 ± 26.5	32.7 ± 29.3	37.2 ± 27.4	32.8 ± 30.2
Peak CK-MB (ng/ml)	177.3 ± 171.9	152.1 ± 160.8	244.7 ± 170.7	209.6 ± 171.2
Peak CK (U/L)	1748.8 ± 1721.3	1597.4 ± 1778.6	2632.7 ± 1837.4	2241.2 ± 1759.3
LVEF (%)	51.8 ± 10.6	52.0 ± 10.7	51.4 ± 12.9	54.4 ± 10.7
BNP (pg/ml)	463.6 ± 529.3	555.5 ± 841.7	423.0 ± 491.1	162.2 ± 156.7^*^

*CMR, cardiac magnetic resonance; SU, sulfonylureas; CK-MB, creatine kinase isoenzymes; CK, creatine kinase; LVEF, left ventricular ejection fraction; BNP, brain natriuretic peptide; SU group compared to non-SU group in total population and SU group compared to non-SU group in CMR population*.

* *p < 0.05*.

### Outcomes in CMR Population

No significant difference was observed between sulfonylurea and non-sulfonylurea users in terms of max cTnI, max CK-MB, max CK, and LVEF, whereas sulfonylurea users had higher BNP level compared to non-sulfonylurea users [mean (SD): 423.0 (491.1) ng/ml vs. 162.2 (156.7) ng/ml, *p* = 0.018] (Table 2). As for CMR imaging results, sulfonylureas use was not associated with larger infarct size when compared with non-sulfonylureas use [mean (SD): 26.5 (13.0) vs. 25.1 (8.5), *p* = 0.632). Similarly, no differences in IMH and other CMR parameters were observed between sulfonylureas and non-sulfonylureas patients. By contrast, the incidence of MVO was 88.0% (22/25) in the sulfonylureas group compared with 62.5% (25/40) in the non-sulfonylureas group, indicating that sulfonylureas increased risk for MVO (*p* < 0.05; Table 3).

**Table 3:** Association of sulfonylurea use with CMR imaging results.

**Variables**	**SU group**	**Non-SU group**
	**(*n* = 25)**	**(*n* = 40)**
Infarct size, % of LVM	26.5 ± 13.0 (24)	25.1 ± 8.5 (36)
Microvascular obstruction, *n* (%)	22 (88.0)	25 (62.5)^*^
Intramyocardial hemorrhage, *n* (%)	18 (72.0)	21 (52.5)
LVM index	71.3 ± 29.8 (25)	78.0 ± 31.1 (39)
LVEDV index	70.5 ± 20.3 (24)	66.5 ± 10.5 (36)
LVESV index	35.0 ± 17.2 (24)	31.4 ± 10.1 (36)
Cardiac output index	2.6 ± 1.0 (23)	2.5 ± 0.5 (34)
**Culprit vessel**, ***n*** **(%)**		
Left anterior descending artery	16 (64.0)	24 (60.0)
Left circumflex artery	2 (8.0)	3 (7.5)
Right coronary artery	7 (28.0)	13 (32.5)
Multi-vessel disease, *n* (%)	14 (56.0)	26 (65.0)
Thrombus aspiration, *n* (%)	8 (32.0)	6 (14.6)
**Baseline TIMI flow grade**, ***n*** **(%)**		
0/1 grade	2 (8.0)	1 (2.4)
2 grades	0 (0.0)	1 (2.4)
≥3 grades	23 (92.0)	38 (95.0)
Stent number	1.2 ± 0.5 (25)	1.2 ± 0.5 (40)
Stent length, mm	30.9 ± 12.3 (25)	31.5 ± 11.5 (37)
Stent diameter, mm	3.3 ± 0.4 (25)	3.1 ± 0.4 (37)

*CMR, cardiac magnetic resonance; LVM, left ventricular mass; LVEDV, left ventricular end diastolic volume; LVESV, left ventricular end systolic volume; TIMI, thrombolysis in myocardial infarction*.

* *p < 0.05*.

### Comparison of Outcomes Between Total Population and CMR Population

Compared with the total population, higher cardiac enzymes (CK and CK-MB) were observed in CMR patients. By contrast, a lower BNP level was found in CMR patients than in total patients (Supplementary Table 4).

### Thirty Days and 6 Months Follow-Ups

The clinical outcomes during follow-up are presented in Table 4. Overall, outcomes at 30 days follow-up were consistent with those at 6 months follow-up. No significant difference was observed between sulfonylurea and non-sulfonylurea users in terms of MACEs, individual ischemic events, and bleeding events. However, the incidence of heart failure was 28.0% (7/25) in the sulfonylureas group compared with 2.5% (1/40) in the non-sulfonylureas group at 6 months follow-up (*p* = 0.004).

**Table 4:** Clinical outcomes in CMR population during follow-up.

**Outcomes**	**SU-group**	**Non-SU group**
	**(*n* = 25)**	**(*n* = 40)**
**30 days follow-up**		
MACE, *n* (%)	1 (4.0)	1 (2.5)
Cardiovascular death, *n* (%)	0 (0)	0 (0)
Recurrent myocardial infarction, *n* (%)	0 (0)	0 (0)
Stroke, *n* (%)	0 (0)	1 (2.5)
Recurrent angina, *n* (%)	1 (4.0)	0 (0)
Revascularization, *n* (%)	0 (0)	1 (2.5)
Heart failure, *n* (%)	6 (24.0)	1 (2.5)^*^
Bleeding, *n* (%)	2 (8.0)	1 (2.5)
**6 months follow-up**		
MACE, *n* (%)	1 (4.0)	2 (5.0)
Cardiovascular death, *n* (%)	0 (0)	0 (0)
Recurrent myocardial infarction, *n* (%)	0 (0)	0 (0)
Stroke, *n* (%)	0 (0)	1 (2.5)
Recurrent angina, *n* (%)	1 (4.0)	0 (0)
Revascularization, *n* (%)	0 (0)	0 (0)
Heart failure, *n* (%)	7 (28.0)	1 (2.5)^**^
Bleeding, *n* (%)	2 (8.0)	0 (0)

*MACE, major adverse cardiovascular events, including occurrence of cardiovascular death, non-fatal myocardial infarction (MI), non-fatal stroke and unstable angina; CMR, cardiac magnetic resonance. Heart failure means occurrence of heart failure during the follow-up durations*.

* *p < 0.05 and*

** *p < 0.01*.

### Risk Factors Associated With Infarct Size, MVO, and Heart Failure

Multivariable linear regression model confirmed that sulfonylurea use was not associated with infarct size, while max CK (β coefficient = 0.394; *p* < 0.001) positively and LVEF% (β coefficient = −0.579; *p* < 0.001) negatively correlated with infarct size (Supplementary Table 5). In addition, multivariable logistic regression model identified that hyperlipemia [odds ratio (OR), 4.074; 95% CI, 1.221–13.591; *p* = 0.022] and sulfonylurea use (OR, 4.645; 95% CI, 1.124–19.198, *p* = 0.034) were statistically associated with the incidence of MVO (Table 5 and Supplementary Table 6). The consistent result was also found when other antidiabetic agents (metformin and acarbose) or symptom to balloon time were considered necessary for a variable to be entered into the multivariable analysis model (Supplementary Table 7). Notably, no dependent risk factors were detected for the risk of heart failure (*p* > 0.05 for each variable at 30 days and 6 months follow-ups in the single and multivariable logistic regression model; Table 5 and Supplementary Tables 8–11).

**Table 5:** Risk factors associated with microvascular obstruction and heart failure assessed by multiple logistic regression analysis.

**Risk factors**	**OR**	**95%CI**	***p***
**Microvascular obstruction**			
Hyperlipemia	4.074	1.221–13.591	**0.022**
Sulfonylureas	4.645	1.124–19.198	**0.034**
**Heart failure at 30 days**			
Max CK	1.001	1.000–1.001	0.120
Max TNI	1.005	0.955–1.058	0.845
LVEF%	0.973	0.870–1.087	0.623
BNP	1.002	0.998–1.007	0.257
Sulfonylureas	5.658	0.487–65.746	0.166
**Heart failure at 6 months**			
Max CK	1.001	1.000–1.001	**0.034**
LVEF%	0.953	0.857–1.059	0.372
BNP	1.002	0.998–1.005	0.374
Sulfonylureas	9.630	0.854–108.584	0.067

*CK, creatine kinase; TNI, troponin I; LVEF, left ventricular ejection fraction; BNP, brain natriuretic peptide; RR, Relative Risk; CI, confidence interval*.

*p < 0.05 were considered as statistically different. The bold values indicates significantly difference*.

## Discussion

Our study's major novelty was to investigate the association between sulfonylureas use and infarct size in patients with T2DM and STEMI by CMR techniques, which could accurately determine cardiac infarct size. Overall, sulfonylureas use was not associated with larger infarct size compared to non-sulfonylureas use in the CMR population. Myocardial enzymology indexes between sulfonylurea users and non-sulfonylurea users were also similar in both total population and CMR population. However, the use of sulfonylureas might bring about a higher risk of MVO in T2DM patients with STEMI. Indeed, it is essential to acknowledge that our results did not involve glibenclamide (with a relatively high affinity for vascular and cardiac SUR) and therefore cannot be extrapolated to the overall population allocated to sulfonylureas while it is used only in patients treated with gliclazide, glipizide, and glimepiride included in the present analysis.

Up to now, several retrospective studies have been conducted to assess the risk of adverse cardiovascular events with sulfonylureas therapy. An earlier study has found no significant differences in mortality or CK increments between 76 sulfonylurea users and 89 non-sulfonylurea users (17). Although these findings were consistent with our study, the small sample size might limit the ability to assess significant differences between groups. In 2016, Abdelmoneim et al. reported a larger infarct size with sulfonylureas use (18). This study involved a sufficient sample size of 560 patients and adjusted the baseline characteristics, but it had several significant limitations: only used myocardial enzymology indexes (max TnI and CK) as primary outcomes, no data of left ventricular function, and lack of more reliable methods to quantify infarct size. Recently, a retrospective cohort study, including 174,882 diabetes patients that compared sulfonylureas with metformin-persistent monotherapy users reported a higher risk of MACEs (19). Notably, the comparison of metformin has been proven to have protective effects of the cardiovascular system, which inevitably may overestimate the risk of adverse cardiovascular events of sulfonylureas. Conversely, two randomized clinical trials (TOSCA. IT trial and CAROLINA trial) have shown that sulfonylureas use was not associated with an increase in risk for adverse cardiovascular events when compared to pioglitazone or linagliptin (11, 12). These two trials make up the limitation of small sample size and strengthen the internal authenticity of results, while there was also a lack of quantitative assessment of infarct size. Given the latter limitations, this study distinguished sulfonylureas users and non-sulfonylureas users in both total population and CMR population to assess the effect of sulfonylureas on cardiac enzymes and on myocardial infarct size in patients with T2DM and STEMI. The baseline characteristics (including demographic data, cardiovascular risk factors, reperfusion management, and coadministration drugs) were relatively comparable between groups. Furthermore, the multivariable regression model was used to explore the potential risk factors associated with infarct size and adverse clinical outcomes.

Insights from cardiac enzymes, no significant difference was observed between sulfonylurea and non-sulfonylurea users in both total population and CMR population. Although myocardial enzymology indexes, such as cTnI and CK-MB, are more specific to the heart than other biomarkers and has a good correlation with CMR imaging in AMI patients (14, 20, 21), high variant and corresponding wide standard deviation of cardiac enzymes indexes may introduce type II errors on statistics, thus leading to the inconclusiveness of results. Unlike myocardial enzymology indexes, CMR can assess myocardial infarct size and the extent of MVO with high spatial resolution and excellent reproducibility, providing high-resolution images of infarcted myocardium by using delayed hyperenhancement. Delayed hyperenhancement after contrast enables accurate delineation between the infracted and viable myocardium, allowing for the prediction of myocardial functional recovery (22). Therefore, CMR imaging is an excellent method to estimate infarct size and the extent of MVO as surrogate endpoints for comparing different treatment strategies. Its higher accuracy allows for sample size reduction (23). In the present study, the CMR result found a lack of association between the use of sulfonylureas and the larger infarct size in patients with T2DM and STEMI, which provided further evidence except for myocardial enzymology indexes. It is worth noting that other oral antidiabetic agents, such as metformin and acarbose, might bring about positive effects on the cardiovascular system. A real-world study, which included 14,306 acarbose users and 196,143 sulfonylurea users, indicated a lower risk of major atherosclerotic events and ischemic stroke of acarbose compared to sulfonylureas (24). To robust the result, metformin and acarbose were considered necessary for a variable to be entered in the multivariable analysis model. The result confirmed that sulfonylurea use was not statistically associated with the infarct size. In addition, symptom-to-balloon time is a strong predictor of adverse events after primary PCI (25). Patients with anterior myocardial infarction may perform worse left heart function than non-anterior myocardial infarction (26). Therefore, we did subgroup analyses by including patients whose symptom-to-balloon time was <12 h or patients with anterior myocardial infarction; the results were consistent with the primary results, thus strengthening our findings. As a prognosticator for morbidity after AMI (27), MVO was proven to be affected by diabetes and dyslipidemia (28). Our multivariable analysis model, including 65 diabetes and CMR imaging patients, found that dyslipidemia and sulfonylureas were the independent factors for MVO. Currently, the pathophysiology of dyslipidemia-associated MVO has not been well-established but likely involves the deterioration of myocardial NO metabolism and enhanced the formation of reactive oxygen species (29, 30). Concerning sulfonylureas, the underlying mechanism is also uncertain. Stable blood glucose control can reduce diabetic microvascular disease, while sulfonylureas increase four- to 5-fold risk of severe hypoglycemia when prescribed as initiating monotherapy (31). Therefore, we speculated that sulfonylureas-related MVO might be correlated to their hypoglycemia risks. Further studies certainly are needed to verify our inference.

Considering clinical outcomes, our negative results may lack enough causal inference because of the limited sample size in the CMR population. Indeed, two large randomized controlled trials (RCTs) have addressed the cardiovascular safety associated with sulfonylureas in T2DM (11, 12, 32). Both TOSCA. IT and CAROLINA trial indicated that sulfonylureas were not associated with a significantly increased risk of composite cardiovascular outcomes than pioglitazone or linagliptin (11, 12). It is important that real-world practice entails a more and representative population, which could supplement and validate the conclusions drawn from RCTs. Patorno et al. conducted a population-based study, which identified 24,131 propensity-score-matched pairs of linagliptin and glimepiride initiators. In addition, they found that no statistically significant differences of primary composite cardiovascular outcomes in the comparison of linagliptin and glimepiride [hazard ratio (HR, 0.91; 95%CI 0.79–1.05] (32). Compared with the above studies, our study confirmed the cardiovascular safety of sulfonylureas from three aspects: myocardial enzyme (enzymology), myocardial infarct size (imaging), and clinical outcomes. The negative results seemed to be reasonable and explicable. Furthermore, increased heart failure risk in sulfonylureas compared with non-sulfonylureas might be due to the differences in BNP's baseline data. It is undeniable that the SU group had significantly higher BNP that may have been an indicator of worse cardiac function in follow-ups. However, further analysis by multiple logistic regression analysis failed to identify sulfonylureas as an independent risk factor related to heart failure. Therefore, the risk of increased heart failure with sulfonylureas use should be further studied.

Our study's major advantage was to use the myocardial enzymes, infarct size, and clinical outcomes to present a comprehensive picture of the relationship between sulfonylureas use and cardiovascular safety in patients with T2DM and STEMI. Indeed, there were some limitations in our study. First, we did not collect data on the duration of sulfonylurea use, the specific doses, or patients' adherence to their medication regimens, which limited the evaluation of the effect on infarct size among subgroups. Second, selection bias is inherent in this retrospective study. Thus, our results may be used only in CMR patients with similar characteristics, and further verification is needed to extrapolate to the whole population. Third, we did not assess the GRACE score in this study, which may influence the results. Fourth, adjusted confounding methods, such as propensity score matching, could not be available as the limited sample size in the CMR population, thus possibly introducing certain biases. Finally, because different SU derivatives vary for their affinity to vascular, cardiac, and pancreatic sulphonylureum receptors, none of the patients using glibenclamide is a limitation to test the original hypothesis that SU derivatives increase reperfusion injury by closing K-ATP channels. Therefore, further studies are needed to verify the results.

## Conclusions

In this study, sulfonylureas use did not associate with larger infarct size compared with non-sulfonylureas use in T2DM patients with STEMI. Potentially increased risks for MVO need to be confirmed by further randomized controlled trials or more extensive real-world studies. Notably, interpretation of the lack of any impact of sulphonylurea use on infarct size should take into account that most patients presented after a relatively long period of ischemia and that glibenclamide was not used by the included patients in this observational study. Since animal research has shown that the most benefit of opening K-ATP channels occurs in reperfusion models of limited ischemia duration (<1 h) and with glibenclamide as a K-ATP blocker, the results of this study should not be extrapolated to clinical settings with short periods of ischemia or to patients using glibenclamide.

## Data Availability Statement

The original contributions presented in the study are included in the article/Supplementary Material, further inquiries can be directed to the corresponding authors.

## Ethics Statement

The study protocol was approved by ethics committees of Renji Hospital, School of Medicine, Shanghai Jiaotong University (KY2019-159). The patients/participants provided their written informed consent to participate in this study.

## Author Contributions

Z-CG and HG are the guarantors of the entire manuscript. Z-CG, F-HS, HL, and L-CK contributed to the study conception and design, critical revision of the manuscript for important intellectual content, and final approval of the version to be published. LS and Y-HJ contributed to the data acquisition, analysis, and interpretation. All authors contributed to the article and approved the submitted version.

## Conflict of Interest

The authors declare that the research was conducted in the absence of any commercial or financial relationships that could be construed as a potential conflict of interest.
