# Supplementary material for: Sulfonylureas Use Is Not Associated With Increased Infarct Size in Patients With Type 2 Diabetes and ST-Segment Elevation Myocardial Infarction
Source: Front Cardiovasc Med. 2021 May 28;8:658059. doi: 10.3389/fcvm.2021.658059 (PMC8194070; doi:10.3389/fcvm.2021.658059)
Supplement: Supplementary file 1 [file Data_Sheet_1.PDF]

Table S1. Baseline characteristics between total population and CMR population

Variables	Total population (n=254)	CMR population (n=65)
Age, years	65.6±10.6	61.3±7.6 **
Male, n (%)	198 (78.0)	57 (87.7)
Body weight, mean ±SD	70.9±10.3	71.0±10.8
BMI, kg/m2	24.7±3.1	24.5±3.0
HbA1c%, mean ±SD	8.0±1.6	7.9±1.8
Diabetic duration, year	8.7±6.5	6.9±4.2 *
**Cardiovascular risk factors**		
Hypertension, n (%)	182 (71.7)	44 (67.7)
Dyslipidemia, n (%)	49 (19.3)	37 (56.9) **
Chronic kidney disease, n (%)	16 (6.3)	1 (1.5)
Myocardial infarction history, n (%)	0 (0.0)	0 (0.0)
Killip class II-IV, n (%)	39 (15.4)	22 (33.8) **
Anterior infarction, n (%)	115 (45.3)	27 (41.5)
**Reperfusion management**		
PCI, n (%)	254 (100.0)	65 (100.0)
Symptom-to-balloon time, hour	12.8±15.6	5.3±3.0**
**Preadmission drugs, n (%)**		
gliclazide	43 (16.9)	16 (24.6)
glipizide	24 (9.4)	7 (10.8)
glimepiride	32 (12.6)	3 (4.6)
gliquidone	3 (1.2)	0 (0.0)
metformin	79(31.1)	23 (35.4)
acarbose	74 (29.1)	32 (49.2) **
voglibose	7 (2.8)	1 (1.5)
insulin	76 (29.9)	9 (13.8) **
TZD	11(4.3)	1(1.5)

other AHA	19 (7.5)	7(10.8)
Calcium channel blocker	71 (28.0)	12 (18.5)
Beta-blocker	28 (11.0)	5 (7.7)
ACEI or ARB	72 (28.3)	6 (9.2) **
Lipid lowering drug	30 (11.8)	14 (21.5)
Antiplatelet agents	75 (29.5)	38 (15.0) **
Anticoagulants	4 (1.6)	2 (3.1)

CMR, cardiac magnetic resonance; SU, sulfonylureas; STEMI, ST-elevation myocardial infarction; BMI, body mass index; HbA1c%, glycosylated hemoglobin; PCI, percutaneous coronary intervention; AHA, anti-hyperglycemic agents; ACEI, angiotensin converting enzyme inhibitors; ARB, angiotensin receptor blocker; *, $p < 0.05$.

Table S2. Baseline characteristics of patients who were anterior infarction or whose symptom-to-balloon time was less than 12 hours

	Anterior infarction		Symptom-to-balloon time < 12h	
**Variables**	**SU group (n=58)**	**Non-SU group (n=65)**	**SU group (n=80)**	**Non-SU group (n=103)**
Age, years	65.8±10.4	63.3±11.1	66.5±10.0	64.5±10.3
Male, n (%)	44 (75.9)	59 (90.8) *	57 (71.3)	89 (86.4) *
Body weight, mean ±SD	69.3±8.9	72.6±12.2	68.6±8.1	72.0±10.9
BMI, kg/m^2	24.0±2.1	25.1±3.9	23.8±2.1	24.9±3.2
HbA1c%, mean ±SD	8.0±1.2	8.1±1.9	8.0±1.7	7.9±1.6
Diabetic duration, year	7.9±4.6	9.3±6.3	7.9±5.2	8.5±6.4
**Cardiovascular risk factors**				
Hypertension, n (%)	39 (67.2)	52 (80.0)	56 (70.0)	77 (74.8)
Dyslipidemia, n (%)	11 (19.0)	15 (23.1)	17 (21.3)	27 (26.2)
Smoking, n (%)	16 (27.6)	18 (27.7)	17 (21.3)	33 (32.0)
Chronic kidney disease, n (%)	1 (1.7)	6 (9.2)	2 (2.5)	6 (5.8)
Myocardial infarction history, n (%)	0 (0)	0 (0)	0 (0)	0 (0)
Killip class II-IV, n (%)	10 (17.2)	14 (21.5)	15 (18.8)	17 (16.5)
Anterior infarction, n (%)	/	/	44 (55.0)	42 (40.8)
**Reperfusion management**				
PCI, n (%)	58 (100)	65 (100)	80 (100)	103 (100)
Symptom-to-balloon time, hour	12.2±13.5	13.3±14.1	5.7±3.1	5.2±2.6
**Preadmission drugs, n (%)**				
gliclazide	24 (41.4)	/	33 (41.3)	/
glipizide	14 (24.1)	/	20 (25.0)	/
glimepiride	19 (32.8)	/	25 (31.3)	/
gliquidone	1 (1.7)	/	2 (2.5)	/
metformin	19 (32.8)	16 (24.6)	24 (30.0)	35 (34.0)

acarbose	14 (24.1)	23 (35.4)	18 (22.5)	38 (36.9) *
voglibose	0 (0)	1 (1.5)	1 (1.3)	3 (2.9)
insulin	3 (5.2)	29 (44.6) **	3 (3.8)	42 (40.8)
TZD	3 (5.2)	4 (6.2)	2 (2.5)	6 (5.8)
other AHA	1 (1.7)	8 (12.3) *	3 (3.8)	12 (11.7)
Calcium channel blocker	13 (22.4)	19 (29.2)	18 (22.5)	32 (31.1)
Beta-blocker	3 (5.2)	9 (13.8)	3 (3.8)	15 (14.6)
ACEI or ARB	15 (25.9)	16 (24.6)	26 (32.5)	30 (29.1)
Lipid lowering drug	6 (10.3)	9 (13.8)	8 (10.0)	15 (14.6)
Antiplatelet agents	22 (37.9)	20 (30.8)	26 (32.5)	33 (32.0)
Anticoagulants	1 (1.7)	2 (3.1)	1 (1.3)	2 (1.9)

CMR, cardiac magnetic resonance; SU, sulfonylureas; STEMI, ST-elevation myocardial infarction; BMI, body mass index; HbA1c%, glycosylated hemoglobin; PCI, percutaneous coronary intervention; AHA, anti-hyperglycemic agents; ACEI, angiotensin converting enzyme inhibitors; ARB, angiotensin receptor blocker; SU group compared to non-SU group in total population and SU group compared to non-SU group in CMR population, * means p<0.05 and ** means p<0.01.

Table S3. Association of sulfonylurea use with cardiac enzymes and heart failure indexes in patients who were anterior infarction or whose symptom-to-balloon time was less than 12 hours

Variables	Anterior infarction		Symptom-to-balloon time <12h	
	SU group (n=58)	non-SU group (n=65)	SU group (n=80)	non-SU group (n=103)
Max Troponin I (ng/ml)	29.7±28.2	30.8±29.0	32.1±27.2	34.5±31.2
Peak CK-MB (ng/ml)	195.0±191.1	134.0±149.5	200.0±181.7	186.8±174.0
Peak CK (U/L)	1830.4±1887.7	1513.8±1859.8	1985.7±1828.9	1954.9±1940.4
LVEF (%)	50.5±11.7	49.5±9.9	51.4±10.9	52.7±10.1
BNP (pg/ml)	535.0±628.6	512.1±679.4	486.3±573.1	354.7±551.7

CMR, cardiac magnetic resonance; SU, sulfonylureas; CK-MB, creatine kinase isoenzymes; CK, creatine kinase; LVEF, left ventricular ejection fraction; BNP, brain natriuretic peptide; SU group compared to non-SU group in total population and SU group compared to non-SU group in CMR population.

Table S4. Association of cardiac enzymes and heart failure indexes between total population and CMR population

Variables	Total population (n=254)	CMR population (n=65)
Max Troponin I	31.1±28.2	34.5±29.0
Max CK-MB	162.1±165.4	220.8±170.5*
Max CK	1657.6±1754.2	2391.7±1785.8**
LVEF, %	52.0±10.6	53.3±11.6
BNP	519.0±734.0	260.0±345.6**

CMR, cardiac magnetic resonance; SU, sulfonylureas; CK-MB, creatine kinase isoenzymes; CK, creatine kinase; LVEF, left ventricular ejection fraction; BNP, brain natriuretic peptide; *, p<0.05.

Table S5. Risk factors for infarct size assessed by multiple linear regression

**Risk Factors**	**β coefficient**	***P* value**
Max CK	0.394	**< 0.001**
LVEF%	-0.579	**< 0.001**
Max Troponin I	0.058	0.591
BNP	0.085	0.456
Sulfonylureas	0.025	0.796
Metformin	0.132	0.177
Acarbose	0.067	0.502
Symptom to balloon time	0.032	0.708

CK, creatine kinase; LVEF, left ventricular ejection fraction; BNP, brain natriuretic peptide

Table S6. Risk factors for microvascular obstruction assessed by single logistic regression analysis

Risk Factors	OR	95%CI	*P* value
**Patients' characteristics**			
Age	1.015	0.943-1.092	0.698
Body weight	0.937	0.884-0.994	**0.032**
BMI	0.796	0.647-0.980	**0.031**
HbA1c%	1.198	0.845-1.698	0.311
Diabetic duration	0.937	0.799-1.100	0.427
**Complicating disease**			
Hypertension	0.745	0.226-2.462	0.630
Hyperlipemia	3.875	1.226-12.248	**0.021**
Angina	1.806	0.510-6.397	0.359
**Drug uses**			
Sulfonylureas	4.400	1.123-17.239	**0.033**
Metformin	1.133	0.360-3.567	0.831
Acarbose	0.440	0.141-1.369	0.156
Insulin	0.417	0.098-1.771	0.236
CCB	0.718	0.187-2.760	0.630
β-receptor inhibitor	1.581	0.165-15.187	0.691
Lipid-lowering drugs	1.528	0.372-6.268	0.556
Antiplatelet drug	1.179	0.394-3.532	0.769
**Reperfusion management**			
Symptom to balloon time	1.025	0.843-1.246	0.808

BMI, body mass index; CCB, calcium channel blocker; OR, odds ratio; CI, confidence interval; $p<0.05$ were considered as statistically different.

Table S7. Risk factors of microvascular obstruction assessed by multiple logistic regression analysis

**Risk Factors**	**OR**	**95%CI**	***P* value**
Hyperlipemia	4.333	1.247-15.053	**0.021**
Sulfonylureas	4.517	1.053-19.382	**0.042**
Metformin	1.219	0.280-5.308	0.792
Acarbose	0.802	0.185-3.469	0.768
Symptom to balloon time	0.994	0.766-1.291	0.966

OR, odds ratio; CI, confidence interval; $p<0.05$ were considered as statistically different.

Table S8. Risk factors for heart failure assessed by single logistic regression analysis (30 days follow up)

Risk Factors	OR	95%CI	*P* value
**Patients' characteristics**			
Male	0.824	0.086-7.889	0.866
Age	1.036	0.936-1.145	0.496
Body weight	0.994	0.924-1.070	0.880
BMI	1.006	0.776-1.306	0.961
HbA1c%	1.096	0.665-1.807	0.718
Diabetic duration	1.062	0.854-1.321	0.586
**Complicating disease**			
Hypertension	1.218	0.216-6.864	0.823
Hyperlipemia	1.010	0.207-4.927	0.990
Angina	3.500	0.704-17.403	0.126
**Drug uses**			
Sulfonylureas	12.316	1.383-109.700	**0.024**
Metformin	1.425	0.290-7.001	0.663
Acarbose	0.373	0.067-2.082	0.261
CCB	0.712	0.078-6.534	0.764
β-receptor inhibitor	2.250	0.215-23.546	0.498
Lipid-lowering drugs	1.533	0.264-8.900	0.634
Antiplatelet drug	1.894	0.339-10.579	0.467
**Cardiac function index**			
Max CK	1.000	1.000-1.001	**0.039**
Max CK-MB	1.002	0.998-1.006	0.299
Max TNI	1.032	1.004-1.061	**0.026**
LVEF%	0.911	0.843-0.984	**0.017**
BNP	1.003	1.001-1.006	**0.015**
**Reperfusion management**			

Symptom to balloon time	1.022	0.804-1.297	0.861

BMI, body mass index; CCB, calcium channel blocker; CK, creatine kinase; CK-MB, Creatine Kinase Isoenzyme-MB; TNI troponin; LVEF, left ventricular ejection fraction; BNP, brain natriuretic peptide; OR, odds ratio; CI, confidence interval; $p<0.05$ were considered as statistically different.

Table S9. Risk factors for heart failure assessed by multiple logistic regression analysis (30 days follow up)

**Risk Factors**	**OR**	**95%CI**	***P* value**
Max CK	1.001	1.000-1.002	0.096
Max TNI	1.008	0.951-1.068	0.790
LVEF%	0.960	0.850-1.084	0.511
BNP	1.003	0.998-1.007	0.265
Sulfonylureas	7.307	0.455-117.256	0.160
Metformin	2.719	0.109-67.943	0.542
Acarbose	0.633	0.042-9.597	0.741
Symptom to balloon time	0.889	0.592-1.336	0.572

CK, creatine kinase; TNI troponin; LVEF, left ventricular ejection fraction; BNP, brain natriuretic peptide; OR, odds ratio; CI, confidence interval; $p<0.05$ were considered as statistically different.

Table S10. Risk factors for heart failure assessed by single logistic regression analysis (6 months follow up)

Risk Factors	OR	95%CI	*P* value
**Patients' characteristics**			
Male	0.353	0.058-2.157	0.259
Age	1.051	0.955-1.155	0.308
Body weight	0.968	0.900-1.041	0.378
BMI	0.936	0.728-1.203	0.605
HbA1c%	1.149	0.729-1.808	0.550
Diabetic duration	1.090	0.890-1.334	0.406
**Complicating disease**			
Hypertension	1.500	0.276-8.149	0.639
Hyperlipemia	0.727	0.165-3.202	0.674
Angina	2.562	0.571-11.502	0.219
**Drug uses**			
Sulfonylureas	15.167	1.734-132.628	**0.014**
Metformin	2.000	0.450-8.886	0.362
Acarbose	0.300	0.056-1.614	0.161
CCB	1.567	0.275-8.924	0.613
β-receptor inhibitor	1.893	0.184-19.430	0.591
Lipid-lowering drugs	1.250	0.223-6.997	0.800
Antiplatelet drug	2.344	0.435-12.622	0.321
**Cardiac function index**			
Max CK	1.000	1.000-1.001	**0.015**
Max CK-MB	1.004	1.000-1.008	0.063
Max TNI	1.023	0.998-1.049	0.072
LVEF%	0.919	0.856-0.987	**0.020**
BNP	1.003	1.000-1.005	**0.020**
**Reperfusion management**			

Symptom to balloon time	1.028	0.825-1.282	0.804

BMI, body mass index; CCB, calcium channel blocker; CK, creatine kinase; CK-MB, Creatine Kinase Isoenzyme-MB; TNI troponin; LVEF, left ventricular ejection fraction; BNP, brain natriuretic peptide; OR, odds ratio; CI, confidence interval; $p<0.05$ were considered as statistically different.

Table S11. Risk factors for heart failure assessed by multiple logistic regression analysis (6 months follow up)

**Risk Factors**	**OR**	**95%CI**	***P* value**
Max CK	1.001	1.000-1.001	**0.036**
LVEF%	0.952	0.849-1.067	0.395
BNP	1.002	0.998-1.006	0.316
Sulfonylureas	15.265	0.793-294.012	0.071
Metformin	3.042	0.192-48.248	0.430
Acarbose	0.474	0.042-5.311	0.545
Symptom to balloon time	0.870	0.617-1.226	0.426

CK, creatine kinase; LVEF, left ventricular ejection fraction; BNP, brain natriuretic peptide; OR, odds ratio; CI, confidence interval; $p<0.05$ were considered as statistically different.
